# Syndrome of inappropriate antidiuretic hormone secretion as a side effect of chemotherapy for testicular cancer: A case report

**DOI:** 10.1002/iju5.12122

**Published:** 2019-10-11

**Authors:** Koki Maeda, Susumu Kageyama, Takashi Osafune, Yoshikata Masuda, Shota Nakagawa, Kenji Miki, Shun Esumi, Maiko Kakita‐Kobayashi, Tetsuya Yoshida, Mitsuhiro Narita, Akihiro Kawauchi

**Affiliations:** ^1^ Department of Urology Uji‐Tokushukai Medical Center Uji Kyoto Japan; ^2^ Department of Urology Shiga University of Medical Science Otsu Shiga Japan; ^3^ Department of Emergency and General Medicine Uji‐Tokushukai Medical Center Uji Kyoto Japan; ^4^ Department of Endocrinology and Metabolism Uji‐Tokushukai Medical Center Uji Kyoto Japan

**Keywords:** syndrome of inappropriate antidiuretic hormone secretion, chemotherapy, hyponatremia, renal salt wasting syndrome, testicular cancer

## Abstract

**Introduction:**

Inappropriate antidiuretic hormone secretion syndrome can be a serious adverse event of cisplatin‐based chemotherapy. Cisplatin had to be changed to other drugs or chemotherapy completely discontinued in earlier reported cases.

**Case presentation:**

Three cycles of bleomycin, etoposide, and cisplatin chemotherapy were planned for a 40‐year‐old man with a diagnosis of lymph node recurrence of testicular cancer. On day 9, he suffered from vomiting and mental disturbance. Severe hyponatremia (110 mEq/L) with low plasma osmolality led to a diagnosis of a syndrome of inappropriate antidiuretic hormone secretion, and infusions of hypertonic saline and salt intake were prescribed. Second and third courses of bleomycin, etoposide, and cisplatin chemotherapy could then be given with careful electrolyte management.

**Conclusion:**

Continuation of cisplatin administration with precise electrolyte adjustment can be a treatment option in regimens where cisplatin is essential for achieving optimal antitumor efficacy.

**Abbreviations & Acronyms**
ADH: antidiuretic hormone
BEP: bleomycin, etoposide, cisplatin
CT: computed tomography
RSWS: renal salt wasting syndrome
SIADH: syndrome of inappropriate antidiuretic hormone secretion

**Keynote message**

Inappropriate antidiuretic hormone secretion syndrome can be a serious side effect of cisplatin‐containing chemotherapy. Continuation of cisplatin administration with precise electrolyte adjustment can be a treatment option in regimens where cisplatin is essential to achieve optimal antitumor effects.

## Introduction

The SIADH is a known serious adverse event associated with cisplatin‐based chemotherapy. In earlier reported cases, cisplatin was changed to another drug or chemotherapy was discontinued due to this side effect. Here, we report a patient with testicular cancer presenting with cisplatin‐induced SIADH, for whom we could continue to administer cisplatin‐containing chemotherapy by carefully maintaining an appropriate sodium level.

## Case presentation

**Figure 1:**
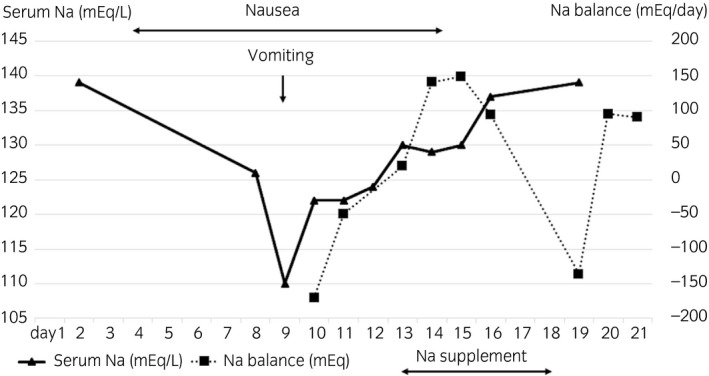
Clinical course and serum sodium level of the first course of chemotherapy.

**Figure 2:**
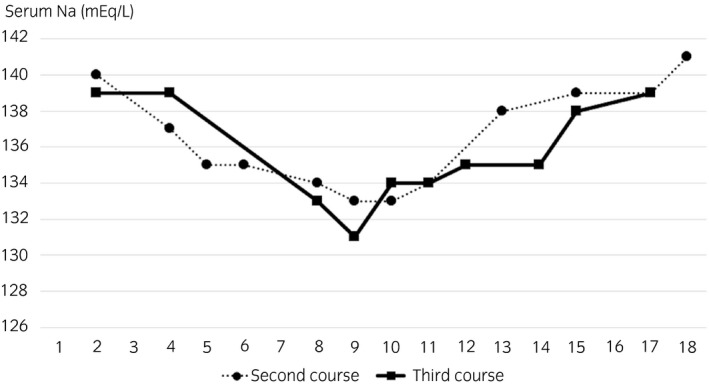
Serum sodium level of the second and the third courses of chemotherapy.

A 40‐year‐old man was admitted to our hospital with a 15 cm right testicular tumor. High inguinal orchiectomy was performed, and pathological diagnosis was seminoma, pT1. A systemic survey showed no metastasis, resulting in assignment to stage I. The patient selected the option of surveillance without adjuvant therapy. One month after surgery, CT showed enlarged pelvic lymph nodes, and the patient was diagnosed as having nodal metastasis. The International Germ Cell Consensus Classification was good prognosis. Therefore three cycles of BEP chemotherapy were planned. The patient complained of nausea and constipation from day 4, without vomiting or loss of consciousness. On day 8, although hyponatremia (Na 126 mEq/L) was observed, gastrointestinal symptoms did not deteriorate, and bleomycin was administered in accordance with the regimen. On day 9, he suffered from vomiting (Common Terminology Criteria for Adverse Events v5.0, Grade 2) and disorientation (Grade 1). Serum sodium level was decreased to 110 mEq/L. Urinary and plasma osmolality were 740 and 231 mOsm/L, respectively. ADH was 10.1 pg/mL (~4.2 pg/mL). Renal, thyroid, and adrenal dysfunctions were excluded. Thus, SIADH was diagnosed. His symptoms gradually improved after administration of 3% hypertonic saline and extra salt intake (Fig. 1). On day 15, febrile neutropenia prevented the administration of bleomycin. The patient recovered from febrile neutropenia with antibiotic treatment. For the second and third chemotherapy courses, serum sodium was frequently monitored (Fig. 2), and hyponatremia was treated by restricting water intake to 800–1000 mL/day and prescribing salt intake of 3.0–4.5 g/day besides meals. Serum sodium was maintained at >130 mEq/L. Mild digestive symptoms without hyponatremia‐related symptoms were recorded. Lymph node metastasis disappeared on CT after three courses of BEP chemotherapy.

## Discussion

SIADH is a serious adverse event associated with chemotherapy regimens containing cisplatin. SIADH was first reported as a syndrome of hyponatremia due to abnormal secretion of ADH by Schwartz *et al*.^1^ Diagnostic criteria include (i) hyponatremia with corresponding hypoosmolality of the serum and extracellular fluid, (ii) continued renal excretion of sodium, (iii) absence of clinical evidence of fluid volume depletion, (iv) urine osmolality greater than that appropriate for the concomitant plasma osmolality, and (v) normal function of kidney, thyroid, and parathyroid.^2^, ^3^, ^4^ All these criteria were fulfilled in the present case. The causes of SIADH include malignant tumors, central nervous system disease, pulmonary disease, and side effects of drugs. Some anticancer agents have been reported to cause SIADH, such as vincristine, vinblastine, cyclophosphamide, and cisplatin.^2^, ^5^, ^6^, ^7^, ^8^, ^9^ The pathogenesis of SIADH caused by cisplatin is still unknown, but it is believed that suppression of Na reabsorption in the ascending limb of Henle in the renal tubules is involved. In patients who undergo chemotherapy, ADH secretion is promoted by vomiting or fluid infusions.^5^, ^8^

The treatment of SIADH is based on hypertonic saline administration and fluid restriction. In acute phase (<48 h after onset), a rapid correction of serum sodium level should be performed. However, in the chronic phase (>48 h after onset), serum sodium elevation should be carefully controlled because there is evidence that rapid overcorrection is associated with a risk of osmotic demyelination syndrome.^3^, ^6^, ^10^ In the present case, after starting to correct serum sodium levels there was an increase to 12 mEq/L per day in the first 24 h. Water was administered freely to adjust the speed of correction, as this hyponatremia was judged as chronic because nausea had already occurred from day 4. Although salt intake is not usually necessary for the treatment of SIADH, it was given in this case to compensate for the increase in urinary sodium excretion.

As chemotherapy‐induced hyponatremia is a serious adverse event, chemotherapy regimens are commonly modified and other drugs such as carboplatin^11^ and nedaplatin^8^ are given, or treatment was changed to surgery or radiation therapy.^9^, ^12^ However, we decided to continue BEP because of a case report of recurrence of SIADH even after switching from cisplatin to carboplatin,^8^ and because cisplatin is probably superior to carboplatin for the treatment of metastatic germ cell tumors.^13^ To the best of our knowledge, there is one case of hyponatremia induced by BEP described in the literature. That case was initially diagnosed with SIADH, but this was changed to RSWS which also causes hyponatremia because of the reabsorption failure of water and sodium due to tubular damage.^14^ Differential diagnosis criteria for SIADH include renal dysfunction, dehydration, and loss of sodium exceeding intake (Table 1).^14^ In the present case, there was no renal dysfunction or dehydration findings, so RSWS was not diagnosed. Serum sodium level was monitored by frequent blood tests after the second course of chemotherapy. Although urinary sodium excretion was increased and hyponatremia was observed during the second and third courses, serum sodium could be maintained at >130 mEq/L (Fig. 2). Hyponatremia induced by cisplatin occurs between several days and 1 week after drug administration in both SIADH and RSWS.^9^, ^12^, ^15^ In the present case, the lowest value was observed on day 9 for all three courses (Fig. 2). The rate of hyponatremia and SIADH of any cause is reported to be approximately 4% and 1%, respectively, in hospitalized cancer patients.^16^ Although SIADH occurs as a side effect of cisplatin in <0.1% of patients according to the product information insert, there have been dozens of reported cases of SIADH and RSWS as a side effect of chemotherapy in the past.^8^, ^9^, ^11^, ^12^, ^15^

**Table 1:** Differential diagnosis of the syndrome of inappropriate antidiuretic hormone secretion and the renal salt wasting syndrome

	SIADH	RSWS	Present case
Hyponatremia	+	+	+
Dehydration	−	+	−
Renal failure	−	+	−
Urinary Na excretion > Na intake	±	+	+
Increased ADH secretion	+	±	+
Treatment	Water restriction	Na supplementation, infusion	Water restriction, Na supplementation

SIADH is a serious adverse event of cisplatin‐containing chemotherapy. Continuation of cisplatin administration with precise electrolyte adjustment can nevertheless remain a treatment option in regimens where cisplatin is essential for the optimal antitumor effects.

## Conflict of interest

The authors declare no conflict of interest.
